# Editorial: Management of acute stroke with large core

**DOI:** 10.3389/fneur.2023.1135886

**Published:** 2023-02-06

**Authors:** Jenny P. Tsai, Joanna D. Schaafsma, Amrou Sarraj

**Affiliations:** ^1^Cerebrovascular Center, Neurological Institute, Cleveland Clinic, Cleveland, OH, United States; ^2^Division of Neurology, Department of Medicine, University of Toronto, Toronto, ON, Canada; ^3^Programs of Neurology and Stroke, University Hospitals Cleveland Medical Center – Case Western Reserve University, Cleveland, OH, United States

**Keywords:** stroke, thrombectomy, large ischemic core, cerebrovascular disease, neuroimaging

Owing to perceived poor outcomes and limited benefit from reperfusion therapies, acute stroke patients presenting with large infarct core on baseline imaging were consistently excluded from thrombolytic and endovascular therapy trials for the last two decades (1–10). Prioritizing finding an effective treatment for the majority of acute stroke patients has paved the way for the current standard-of-care, and improved survival and functional recovery for a vast number of individuals (11, 12). However, whether thrombolytic and endovascular therapies are safe and provide functional benefits in presence of large infarct cores is still unknown. As acute stroke research continues to expand the indication for both medical and endovascular therapies, management of acute stroke patients with large infarct core becomes the next frontier to explore (13–16).

Elucidating the best management of a recently established or evolving large infarct comes down to three key questions. First, what are the optimal imaging modalities and criteria to identify a “large core”? Second, are attempts at reducing the infarct core size *via* thrombolytic or endovascular reperfusion justified by their risk-benefit balance? And third, how can secondary injury and complications of acute large infarcts be minimized?

This review on the topic of acute strokes with large infarct core examines all three aspects. Koopman et al. present evidence that perfusion imaging is a reliable early biomarker to predict clinical outcome in this patient population. Yang et al. continue the discussion with evidence that patient age and perfusion profile may potentially define a ceiling to treatment benefits (17). To address this complexity of early outcome prediction, Chen et al. propose a model based on demographic and imaging biomarkers (18). As the discussion shifts to acute therapy, Hu et al. suggest a promising role for low-dose tirofiban (19). Meanwhile, Hassan et al. present data from the COMPLETE registry to reaffirm the importance of time in limiting infarct growth and improving clinical outcomes (20). DeHoff and Lau then round off our discussion with a review of medical management of cerebral edema resulting from large hemispheric infarcts (21).

The world of cerebrovascular medicine currently eagerly awaits the outcomes of randomized controlled trials on endovascular therapy for acute strokes with large infarct cores (22–24). While endovascular reperfusion may yet again promise the most effective treatment, the complexity of acute stroke patients presenting with large cores highlights the ongoing need for multi-faceted, collaborative research to optimize their outcomes. It is long overdue.

## Author contributions

All authors listed have made a substantial, direct, and intellectual contribution to the work and approved it for publication.

## References

[1] Berkhemer OA Fransen PSS Beumer D van den Berg LA Lingsma HF Yoo AJ. A randomized trial of intraarterial treatment for acute ischemic stroke. N Engl J Med. (2015) 372:11–20. 10.1056/nejmoa1411587 25517348

[2] Campbell BCV Mitchell PJ Kleinig TJ Dewey HM Churilov L Yassi N. Endovascular therapy for ischemic stroke with perfusion-imaging selection. N Engl J Med. (2015) 372:1009–18. 10.1056/nejmoa1414792 25671797

[3] Goyal M Demchuk AM Menon BK Eesa M Rempel JL Thornton J. Randomized assessment of rapid endovascular treatment of ischemic stroke. N Engl J Med. (2015) 372:1019–30. 10.1056/nejmoa1414905 25671798

[4] Saver JL Goyal M Bonafe A Diener H-C Levy EI Pereira VM. Stent-retriever thrombectomy after intravenous t-PA vs. t-PA alone in stroke. N Engl J Med. (2015) 372:2285–95. 10.1056/nejmoa1415061 25882376

[5] Campbell BCV Mitchell PJ Churilov L Yassi N Kleinig TJ Dowling RJ. Tenecteplase versus alteplase before thrombectomy for ischemic stroke. N Engl J Med. (2018) 378:1573–82. 10.1056/nejmoa1716405 29694815

[6] Nogueira RG Jadhav AP Haussen DC Bonafe A Budzik RF Bhuva P. Thrombectomy 6 to 24 hours after stroke with a mismatch between deficit and infarct. N Engl J Med. (2017) 378:11–21. 10.1056/nejmoa1706442 29129157

[7] Albers GW Marks MP Kemp S Christensen S Tsai JP Ortega-Gutierrez S. Thrombectomy for stroke at 6 to 16 hours with selection by perfusion imaging. N Engl J Med. (2018) 378:708–18. 10.1056/nejmoa1713973 29364767 PMC6590673

[8] Jovin TG Chamorro A Cobo E de Miquel MA Molina CA Rovira A. Thrombectomy within 8 hours after symptom onset in ischemic stroke. N Engl J Med. (2015) 372:2296–306. 10.1056/nejmoa1503780 25882510

[9] Ma H Campbell BCV Parsons MW Churilov L Levi CR Hsu C. Thrombolysis guided by perfusion imaging up to 9 hours after onset of stroke. N Engl J Med. (2019) 380:1795–803. 10.1056/nejmoa1813046 31067369

[10] Group NI of ND, Srt-PSS. Tissue plasminogen activator for acute ischemic stroke. N Engl J Med. (1995) 333:1581–88. 10.1056/nejm199512143332401 7477192

[11] Goyal M Menon BK van Zwam WH Dippel DWJ Mitchell PJ Demchuk AM. Endovascular thrombectomy after large-vessel ischaemic stroke: a meta-analysis of individual patient data from five randomised trials. Lancet. (2016) 387:1723–31. 10.1016/s0140-6736(16)00163-x 26898852

[12] Albers GW Lansberg MG Brown S Jadhav AP Haussen DC Martins SO. Assessment of optimal patient selection for endovascular thrombectomy beyond 6 hours after symptom onset: a pooled analysis of the AURORA database. JAMA Neurol. (2021) 78:1064. 10.1001/jamaneurol.2021.2319 34309619 PMC8314176

[13] Garcia-Esperon C Bivard A Johns H Chen C Churilov L Lin L. Association of endovascular thrombectomy with functional outcome in patients with acute stroke with a large ischemic core. Neurology. (2022) 99:e1345–e1355. 10.1212/wnl.0000000000201641 35803723

[14] Moreu M Pérez-García C Rosati S López-Frías A Egido JA Gómez-Escalonilla C. Mechanical thrombectomy is cost-effective versus medical management alone around Europe in patients with low ASPECTS. J Neurointerv Surg. (2022) jnis-2022-019849. 10.1136/jnis-2022-019849. [Epub ahead of print]. 36564198 PMC10313965

[15] Sarraj A Hassan AE Savitz S Sitton C Grotta J Chen P. Outcomes of endovascular thrombectomy vs medical management alone in patients with large ischemic cores: a secondary analysis of the optimizing patient's selection for endovascular treatment in acute ischemic stroke (SELECT) study. JAMA Neurol. (2019) 76:1147. 10.1001/jamaneurol.2019.2109 31355873 PMC6664381

[16] Tanaka K Goyal M Menon BK Campbell BCV Mitchell PJ Jovin TG. Significance of baseline ischemic core volume on stroke outcome after endovascular therapy in patients age ≥75 years: a pooled analysis of individual patient data from 7 trials. Stroke. (2022) 53:3564–71. 10.1161/strokeaha.122.039774 36337054

[17] Yang H Lin D Lin X Wu Y Yi T Chen W. Outcomes and CT perfusion thresholds of mechanical thrombectomy for patients with large ischemic core lesions. Front Neurol. (2022) 13:856403. 10.3389/fneur.2022.856403 35720105 PMC9198314

[18] Chen J Li J Xu Z Zhang L Qi S Yang B. Prediction model of early biomarkers of massive cerebral infarction caused by anterior circulation occlusion: establishment and evaluation. Front Neurol. (2022) 13:903730. 10.3389/fneur.2022.903730 36062018 PMC9433650

[19] Hu Y Xiao Q Shi Z Hou Y Chen Z Cheng J. Safety and efficacy of low-dose and long-course tirofiban in large hemispheric infarction. Front Neurol. (2022) 13:987859. 10.3389/fneur.2022.987859 36158948 PMC9500446

[20] Hassan AE Zaidat OO Nanda A Atchie B Woodward K Doerfler A. Impact of interhospital transfer vs. direct admission on acute ischemic stroke patients: a subset analysis of the COMPLETE registry. Front Neurol. (2022) 13:896165. 10.3389/fneur.2022.896165 36016541 PMC9397115

[21] DeHoff G Lau W. Medical management of cerebral edema in large hemispheric infarcts. Front Neurol. (2022) 13:857640. 10.3389/fneur.2022.857640 36408500 PMC9672377

[22] Sarraj A Hassan AE Abraham M Ribo M Blackburn S Chen M. A randomized controlled trial to optimize patient's selection for endovascular treatment in acute ischemic stroke (SELECT2): study protocol. Int J Stroke. (2021) 17:689–93. 10.1177/17474930211035032 34282987

[23] Jabal MS Ibrahim MK Thurnham J Kallmes KM Kobeissi H Ghozy S. Common data elements analysis of mechanical thrombectomy clinical trials for acute ischemic stroke with large core infarct. Clin Neuroradiol. (2022) 1–11. 10.1007/s00062-022-01239-x. [Epub ahead of print]. 36520186

[24] Yoshimura S Sakai N Yamagami H Uchida K Beppu M Toyoda K. Endovascular therapy for acute stroke with a large ischemic region. N Engl J Med. (2022) 386:1303–13. 10.1056/nejmoa2118191 35138767

